# A Generator-Produced Gallium-68 Radiopharmaceutical for PET Imaging of Myocardial Perfusion

**DOI:** 10.1371/journal.pone.0109361

**Published:** 2014-10-29

**Authors:** Vijay Sharma, Jothilingam Sivapackiam, Scott E. Harpstrite, Julie L. Prior, Hannah Gu, Nigam P. Rath, David Piwnica-Worms

**Affiliations:** 1 BRIGHT Institute, Molecular Imaging Center, Mallinckrodt Institute of Radiology, Washington University School of Medicine, St. Louis, Missouri, United States of America; 2 Department of Biomedical Engineering, Washington University School of Medicine, St. Louis, Missouri, United States of America; 3 Departments of Cell Biology and Physiology and Developmental Biology, Washington University School of Medicine, St. Louis, Missouri, United States of America; 4 Department of Chemistry and Biochemistry, University of Missouri, St. Louis, Missouri, United States of America; Stanford University School of Medicine, United States of America

## Abstract

Lipophilic cationic technetium-99m-complexes are widely used for myocardial perfusion imaging (MPI). However, inherent uncertainties in the supply chain of molybdenum-99, the parent isotope required for manufacturing ^99^Mo/^99m^Tc generators, intensifies the need for discovery of novel MPI agents incorporating alternative radionuclides. Recently, germanium/gallium (Ge/Ga) generators capable of producing high quality ^68^Ga, an isotope with excellent emission characteristics for clinical PET imaging, have emerged. Herein, we report a novel ^68^Ga-complex identified through mechanism-based cell screening that holds promise as a generator-produced radiopharmaceutical for PET MPI.

## Introduction

Cardiovascular disease remains the leading cause of death in developed countries as well as in most developing countries [1]. Myocardial perfusion imaging (MPI), a non-invasive measure of blood flow in the heart, is commonly used to determine areas of reversible ischemia, characterize infarcted tissue, and assess left ventricular function [2]. Currently, single photon emission computed tomography (SPECT) imaging with the radioactive transition metal technetium-99m (^99m^Tc; t_½_  = 6.2 hrs) incorporated into monocationic complexes (e.g., ^99m^Tc-sestamibi, ^99m^Tc-tetrofosmin) is widely used [2], [3]. Following intravenous injection, these ^99m^Tc-complexes distribute into heart tissue in proportion to blood flow and remain trapped for times sufficient to image perfusion territories. Molybdenum-99 (^99^Mo; t_½_  = 66 hrs), the parent isotope of ^99m^Tc, is produced by nuclear fission in reactors from enriched uranium-235, and is packaged into ^99^Mo/^99m^Tc generators for distribution to nuclear pharmacies. This production and distribution model is convenient, cost effective, and has provided on-site formulation advantages for decades. However, there are only five active production reactors in the world [4], [5], and healthcare disruptions during the ^99^Mo/^99m^Tc crisis of 2008–2010, in which only two reactors were on-line [6], demonstrated the vulnerability of the world supply of ^99^Mo. Furthermore, proposals for upgrades of these reactors are relatively expensive, and security concerns have been raised for the continued dependence on a technology requiring enriched uranium fuel [7]. To address these shortcomings in the supply chain and security, alternative production methodologies for Mo and Tc have been sought [8]. A notable recent advance has been the high yield direct production of ^99m^Tc by proton-bombardment of ^100^Mo using a conventional medical cyclotron [8]. Although commercialization of these methodologies could substantially lower overall dependence upon enriched uranium and security concerns associated with handling of processed nuclear waste, the high costs of various alternative production strategies complicate medical reimbursement models. Thus, there is need for novel MPI agents derived from alternative radionuclides, particularly agents incorporating isotopes compatible with positron emission tomography (PET) imaging, a technology with superior spatial and temporal resolution compared to SPECT [2], [9].

MPI agents based on flourine-18 (^18^F; t_½_  = 110 min), a common cyclotron-produced PET isotope, have been reported [10], [11], but ^18^F-based agents depend on the presence of nearby cyclotrons, which place constraints on isotope distribution logistics. Another PET MPI agent is rubidium-82 chloride (t_½_  = 75 sec), obtained via electron capture from strontium-82 (t_½_  = 25 d), but the high cost and short half-life of this generator-produced isotope limit its utility. Recently, germanium/gallium (Ge/Ga) generators capable of producing high quality ^68^Ga (t_½_  = 68 min), an isotope with excellent emission properties for clinical PET imaging [12], have emerged. The parent isotope, ^68^Ge (t_½_  = 271 days), is produced in high energy proton accelerators from a ^69^Ga(p,2n)^68^Ge reaction and is bonded to alumina for eventual elution on-site [13], thus providing a practical generator-based distribution model for on-site formulation of PET radiopharmaceuticals. With co-development of high quality ^68^Ga-based tracers, PET imaging could be unlinked from proximity to cyclotrons, thereby expanding access to the technology. A variety of synthetic cationic complexes of Ga(III) have been reported as candidate ^68^Ga PET tracers [14]–[23]; however, identification of a ^68^Ga-complex with biochemical and pharmacokinetic properties ideally suited for PET MPI has proven elusive.

## Materials and Methods

### General Methods

All reagents were purchased from Sigma-Aldrich unless otherwise stated. The linear tetramine, 1,2-ethylenediamino-bis(2,2-dimethylaminopropane) was synthesized as described previously [20]. ^1^H NMR and proton-decoupled ^13^C NMR spectra were recorded on either a Varian (300 MHz) or Bruker (400 MHz) spectrometer; chemical shifts are reported in δ (ppm) with reference to TMS. Mass spectra were obtained from the Washington University Resource for Biomedical and Bioorganic Mass Spectrometry using samples diluted in 50/50 methanol/water containing 0.1% formic acid and analyzed via HRESI. Elemental analyses were performed by Galbraith Laboratories, Knoxville, TN. HPLC analysis was performed with a Waters System 600 equipped with dual λ-detector 2487 (set to 280 and 214 nm) and a γ-detector (Bioscan) for identification of radiopeaks. Gallium(III) complex **4** and its radiolabeled analogues **5a** and **5b** were assessed for purity on a C-18 reversed-phase column (Vydac TP, 10 µm, 300 Å) using an eluent gradient of ethanol and saline (isocratic 20% ethanol in saline for 5 min; gradient from 20% to 90% ethanol in saline from 5–40 min, at a flow of 2 mL/min). Radiochemical purity was determined on C-18 plates employing a mobile eluent mixture of 90% ethanol in saline, using a radio-TLC (Bioscan System 200 Image Scanner). Metabolite analysis was performed using radio-TLC (Bioscan System 200 Image Scanner).

### Chemical Synthesis

#### 2-hydroxy-3-isopropoxy-benzaldehyde (2)

2-isopropoxyphenol **1** (1.34 mmol), anhydrous magnesium chloride (6.73 mmol), and anhydrous triethylamine (13.4 mmol) were suspended in anhydrous acetonitrile (50 mL), and the suspension stirred for 1 h at room temperature. Then, *p*-formaldehyde (6.72 mmol) was added to the mixture and the contents were heated at reflux for 4 h. The reaction mixture was cooled to room temperature, hydrolyzed, acidified with 10% HCl (50 mL), and extracted with ether (3×200 mL). The combined organic extract was dried over anhydrous sodium sulfate, filtered, concentrated, and the residue was purified on silica gel GF254 (Analtech, USA) using a hexane/ethyl acetate (70/30) eluent mixture; 57% yield. ^1^H NMR (300 MHz, CDCl_3_) δ: 1.38 (d, 6H), 4.58 (sept, 1H), 6.93 (t, 1H), 7.13–7.20 (dd, 2H), 9.91 (s, 1H), 10.97 (s, 1H); ^13^C NMR (75 MHz, CDCl_3_) δ: 22.3, 72.4, 119.8, 121.6, 123.1, 125.5, 146.7, 153.2, 196.7; MS(HRESI) Calcd for [C_10_H_12_O_3_]^+^: 163.0754; found: 163.0759.

#### 3,3′-(2-hydroxy-3-isopropoxyphenylimidazolidine-1,3-diyl)bis[1-{(2-hydroxy-3-isopropoxyphenyl)methyleneamino-2,2-dimethyl}propane] (3)

To obtain **3**, starting precursors, 2-hydroxy-3-isopropoxy-1-benzaldehyde **2** (2.7 mmol) and 1,2-ethylenediamino-bis(2,2-dimethylaminopropane) (0.90 mmol) were dissolved in ethanol (10 mL), refluxed for 45 min, and purified by methods described previously [20], [22] . ^1^H NMR (400 MHz, CDCl_3_) δ: 0.79 (s, 12H), 1.27 (d, 6H), 1.36 (d, 12H), 2.27 (d, 2H), 2.52 (d, 2H), 2.64 (s, 2H), 3.0 (d, 2H), 3.38 (d, 2H), 3.50 (d, 2H) 3.74 (s, 1H), 4.40 (sept, 1H), 4.55 (sept, 2H), 6.60 (br, d, 2H), 6.71–6.90 (m, 7H), 8.05 (s, 2H), 10.20 (s, 1H), 13.9 (s, 2H); ^13^C NMR (100 MHz, CDCl_3_) δ: 22.3, 22.4, 24.6, 24.8, 36.7, 54.6, 62.6, 68.1, 71.2, 72.3, 91.8, 117.6, 118.1, 118.3, 119.2, 119.6, 123.6, 124.7, 146.2, 146.6, 149.7, 153.5, 165.5. MS(HRESI), Calcd for [C_42_H_60_N_4_O_6_]: 716.4513; found: [M+H]^+^717.4598.

#### (1,2-ethylenediamino-bis[1-{(3-isopropoxyphenyl-2-ate)methyleneamino-2,2-dimethyl}-propane]gallium(III))iodide [ENBDMP-3-isopropoxy-PI-Ga]+ I− (4)

The ligand **3** (100 mg, 0.18 mmol) dissolved in methanol (5 mL) was treated with dropwise addition of gallium(III) acetylacetonate (66.2 mg, 0.18 mmol) dissolved in methanol. The contents were refluxed for 3 h. Then, potassium iodide (30 mg, 0.18 mmol) dissolved in hot water (0.5 mL) was added and the reaction mixture was refluxed further for 15 min, brought to room temperature slowly, and slow evaporation over several days yielded crystalline material; 30% yield. ^1^H NMR (300 MHz, DMSO-*d*_6_) δ: 0.79 (s, 6H), 0.96 (s, 6H), 1.30–1.33 (dd, 12H), 2.63 (d, 2H), 2.79 (d, 4H), 2.94 (br, s, 2H), 3.61–3.75 (m, 4H), 4.63 (sept, 2H), 4.79 (br, s, 2H), 6.62 (t, 2H), 6.87 (d, 2H), 7.04 (d, 2H), 8.18 (s, 2H); ^13^C NMR (300 MHz, DMSO-*d*_6_) δ: 22.0, 22.1, 22.2, 26.2, 35.6, 47.7, 59.2, 68.9, 69.5, 115.7, 119.2, 119.5, 125.8, 148.7, 158.1, 170.3. MS(HRESI) Calcd for [C_32_H_48_N_4_O_4_Ga]^+^: 621.2926, found: *m*/*z* = 621.2930; and Calcd for [^13^C_32_H_48_N_4_O_4_Ga]^+^: 622.2959, found: *m*/*z* = 622.2967. Elemental analysis calculated for C_32_H_48_N_4_O_4_Ga+CH_4_O: C 50.72; H 6.71; N 7.17; Ga 8.92%. Found: C 50.51; H 6.68; N 7.08; Ga 9.05%.

#### Preparation of ^67^Ga-metalloprobe (5a)

Radiolabeled ^67^Ga-metalloprobe (**5a**) was synthesized by following a procedure described earlier with slight modifications [20], [22]. ^67^Ga was obtained as a commercial citrate salt in water (Triad Isotopes), converted into chloride using HCl (6N), extracted in ether (2×2 mL), evaporated, and the residue was treated with acetylacetone to obtain ^67^Ga(acetylacetonate)_3_. The radiolabeled ^67^Ga-metalloprobe was obtained through a ligand exchange reaction involving ^67^Ga(acetylacetonate)_3_ and heptadentate Schiff-base ligand (**3**) dissolved in ethanol at 100°C for 40 min. Alternatively, ^67^Ga-citrate was treated with HCl, loaded on a cation-exchange cartridge (Strata), washed with water (3×3 mL), and eluted with 400 µL of 98% acetone/HCl (0.02 M). Thereafter, HEPES buffer (pH 5.45, 400 µL) was added to the eluent mixture, the pH was adjusted to 4.5, mixed with the ligand solution, and heated at 100°C for 40 min. Reactions were followed using thin-layer chromatography plates (C-18) employing a radiometric scanner (Bioscan), using an eluent mixture of ethanol/saline (90/10; R_f_: 0.23). Finally, ^67^Ga-metalloprobe **5a** was purified by radio-HPLC on a C-18 reversed-phase column (Vydac TP, 10 µm, 300 Å), using the gradient eluent mixture of ethanol and saline described above. The fraction eluting at a retention time of 27.0 min (**5a**) was collected, concentrated, and employed for bioassays.

#### Preparation of ^68^Ga-metalloprobe (5b)

Radiolabeled ^68^Ga-metalloprobe (**5b**) was synthesized by following a procedure described previously with slight modifications [21], [22], [24]. ^68^Ga was obtained from a generator (Eckert & Ziegler Eurotope) as its chloride salt, converted into ^68^Ga(acetylacetonate)_3_ by reacting with acetylacetone (0.01% solution in ethanol) using standard procedures. The radiolabeled ^68^Ga-metalloprobe was obtained through a ligand exchange reaction involving ^68^Ga(acetylacetonate)_3_ and heptadentate Schiff-base precursor (**3**) dissolved in ethanol at 100°C for 20 min. The reaction was followed using thin-layer chromatography as described above. Finally, ^68^Ga-metalloprobe **5b** was purified by radio-HPLC using a C-18 reversed-phase column and the same gradient eluent mixture of ethanol and saline described above. The fraction eluting at a retention time of 27.0 min (**5b**) was collected, concentrated, and employed for bioassays.

#### X-ray Crystallography

Crystals suitable for X-ray crystallography were grown by dissolving **4** in refluxing methanol, slowly bringing the solution to room temperature and then slow evaporation of the methanol solution overnight. A single crystal with approximate dimensions 0.36×0.33×0.21 mm^3^ was mounted on a glass fiber in a random orientation. Preliminary examination and data collection were performed using a Bruker Kappa Apex II (Charge Coupled Device (CCD) Detector system) single crystal X-Ray diffractometer, equipped with an Oxford Cryostream LT device. All data were collected using graphite monochromated Mo Kα radiation (λ = 0.71073 Å) from a fine focus sealed tube X-Ray source. Preliminary unit cell constants were determined with a set of 36 narrow frame scans. The collected data set consisted of combinations of ϖ and φ scan frames with a scan width of 0.5° and counting time of 15 seconds/frame at a crystal to detector distance of 4.0 cm. The collected frames were integrated using an orientation matrix determined from the narrow frame scans. Apex II and SAINT software packages were used for data collection and data integration. Analysis of the integrated data did not show any decay. Final cell constants were determined by global refinement of xyz centroids of 9377 reflections from the complete data set. Collected data were corrected for systematic errors using SADABS [25] based on the Laue symmetry using equivalent reflections. Structure solution and refinement were carried out using the SHELXTL- PLUS software package. The structure was solved by direct methods and refined successfully in the orthorhombic space group, Pbca. Full matrix least-squares refinement was carried out by minimizing Σw(F_o_^2^–F_c_^2^)^2^. The non-hydrogen atoms were refined anisotropically to convergence. The N-H hydrogens were located and refined with geometrical restraints (**Figure S1**). Other hydrogen atoms were treated using appropriate riding model (AFIX m3). Both isopropoxy groups exhibit positional disorder (**Figure S2**). The disorder was resolved by partial occupancy atoms in both chains and the relative occupancies were refined. Two chains were located for the group O3, C25–C27 and the occupancies were refined to 77∶23%. Furthermore, carbon atoms (C28–C30) of the second isopropoxy substituent were also split into two groups with relative occupancies of 63∶37%. These disordered groups were refined with geometrical and displacement parameter restraints. The compound crystallizes with a molecule of H_2_O and a partial methanol as a solvent of crystallization (**Figure S2**). Further, the occupancy of the methanol was refined to ∼19%. The solvent atoms were also refined with geometrical and displacement parameter restraints. X-ray crystallographic data for **4** (crystal data and structure refinement parameters, atomic coordinates, inter-atomic distances and angles, anisotropic displacement parameters, hydrogen coordinates, and torsion angles) are included in **Tables S1–S6**.

### Bioassays

#### Cell Culture

Rat cardiomyoblasts (H9c2) were grown in DMEM (high glucose) supplemented with L-glutamine (2 mM) and heat-inactivated fetal calf serum (10%). Human epidermoid carcinoma KB-3-1 cells and colchicine-selected KB-8-5 cells were grown as previously described [26]. Detailed growth conditions for stably transfected MCF-7 and MCF-7/*MDR1* cells have been described earlier [27]. Briefly, KB-3-1 and KB-8-5 cells were grown in DMEM supplemented with L-glutamine (2 mM) and heat-inactivated fetal calf serum (10%) in the presence of 0 and 10 ng/ml colchicine, respectively. MCF-7 and MCF7/*MDR1* cells were grown in DMEM supplemented with L-glutamine (2 mM) and heat-inactivated fetal calf serum (10%) in the presence of 1 mg/mL G418.

#### Western Blots

Pgp was detected in enriched membrane fractions of cells with monoclonal antibody C219 (Signet Laboratories, Inc., Dedham, MA) as described previously [28].

#### Cell Transport Studies

Cellular transport studies for **5a** were performed in 24-well tissue culture treated plates. Cells (100,000/well) were plated in media and allowed to recover overnight. Media was removed from cells and replaced with media containing the desired concentrations of **5a** (74 kBq/mL) using control buffer either in the absence or presence of MDR modulator LY335979 (1 µM) or 130 mM K^+^/valinomycin (1 µg/mL) buffer [29]. Cells were allowed to incubate under normal incubation conditions (37°C, 5% CO_2_ atmosphere) for 90 min, and then washed 3× with 4°C Solution A (phosphate buffered saline lacking CaCl_2_ and MgCl_2_). Cells were then extracted in 1% sodium dodecyl sulfate with 10 mM sodium borate. Aliquots of the loading solution and ^67^Ga-complexes **5a** stock solutions also were obtained for standardizing cellular data with the extracellular concentration of **5a**. All cell extracts, ^67^Ga-complex **5a** stock solutions, and loading solution samples were assayed for γ-activity in a well-type sodium iodide γ-counter (Cobra II; Packard). Protein mass was estimated by the bicinchoninic acid analysis (Pierce Chemical Co.), using bovine serum albumin as the protein standard. Data are reported as fmol Ga-complex **5a** (mg protein)^−1^ (nM_0_)^−1^ as previously described [20], [24], with nM_0_ representing the total concentration of ^67^Ga-complex in the extracellular buffer.

#### Biodistribution Studies

All animal procedures were approved by the Washington University Animal Studies Committee. Distribution of ^67^Ga-complex **5a** in tissues of male 4 week old FVB wild-type (WT) and *mdr1a/1b^(−/−)^* gene-deleted (KO) mice (Taconic) was determined as previously described [20]. Male 7 week old Sprague-Dawley rats (Harlan) were similarly used. ^67^Ga-Complex **5a** dissolved in ethanol was diluted in saline/ethanol (90/10) to the final concentration (mice: 1,480 kBq/mL; rats: 2,405 kBq/mL). All animals were anesthetized by isofluorane inhalation and injected with radiotracer (mice: 148 kBq, 100 µL; rats: 481 kBq, 200 µL) via bolus injection through a tail vein. Animals were sacrificed by cervical dislocation at 5, 15 (mice) or 30 (rats), 60, and 120 min after injection (*n* = 3 each). Blood samples were obtained by cardiac puncture, organs then harvested rapidly, and all tissue samples analyzed for γ-activity. Data are expressed as the percentage injected dose (%ID) per gram of tissue (tissue kBq (injected kBq)^−1^ (g tissue)^−1^×100).

#### Metabolic Stability

For metabolic stability studies, three 10 week old Sprague Dawley rats were anesthetized by isofluorane inhalation and injected with radiotracer **5a** (81,400 kBq, 220 µL) via bolus injection through a tail vein, sacrificed at 30 min, 60 min, 90 min and blood, urine, and tissue samples were collected as described above. Urine samples were obtained via either bladder puncture or external collection. Tissues were sonicated, suspended in saline/ethanol (70/30), and extracts were analyzed with a radioTLC scanner on C-18 silica gel plates using saline/methanol (90/10) as the eluent mixture. All samples were compared with the parental control and normalized to percentage of parental compound for analysis.

#### MicroPET Imaging

Imaging was performed with 11 week old Sprague-Dawley rats 60 min post intravenous tail-vein injection (22,200 kBq) of HPLC-purified ^68^Ga-radiopharmaceutical **5b** (95/5 saline/ethanol) using a Focus 220 microPET or Inveon PET/CT scanner (Siemens Medical Solutions). For imaging, rats were anesthetized with isoflurane via an induction chamber and maintained with a nose cone. Following anesthesia, the rats were secured in a supine position and placed in an acrylic imaging tray. PET imaging consisted of a 10 min acquisition. Image data were reconstructed into a single frame using standard methods. For anatomical visualization, PET images were also co-registered with CT images from an Inveon PET/CT scanner.

## Results and Discussion

**Figure 1:**
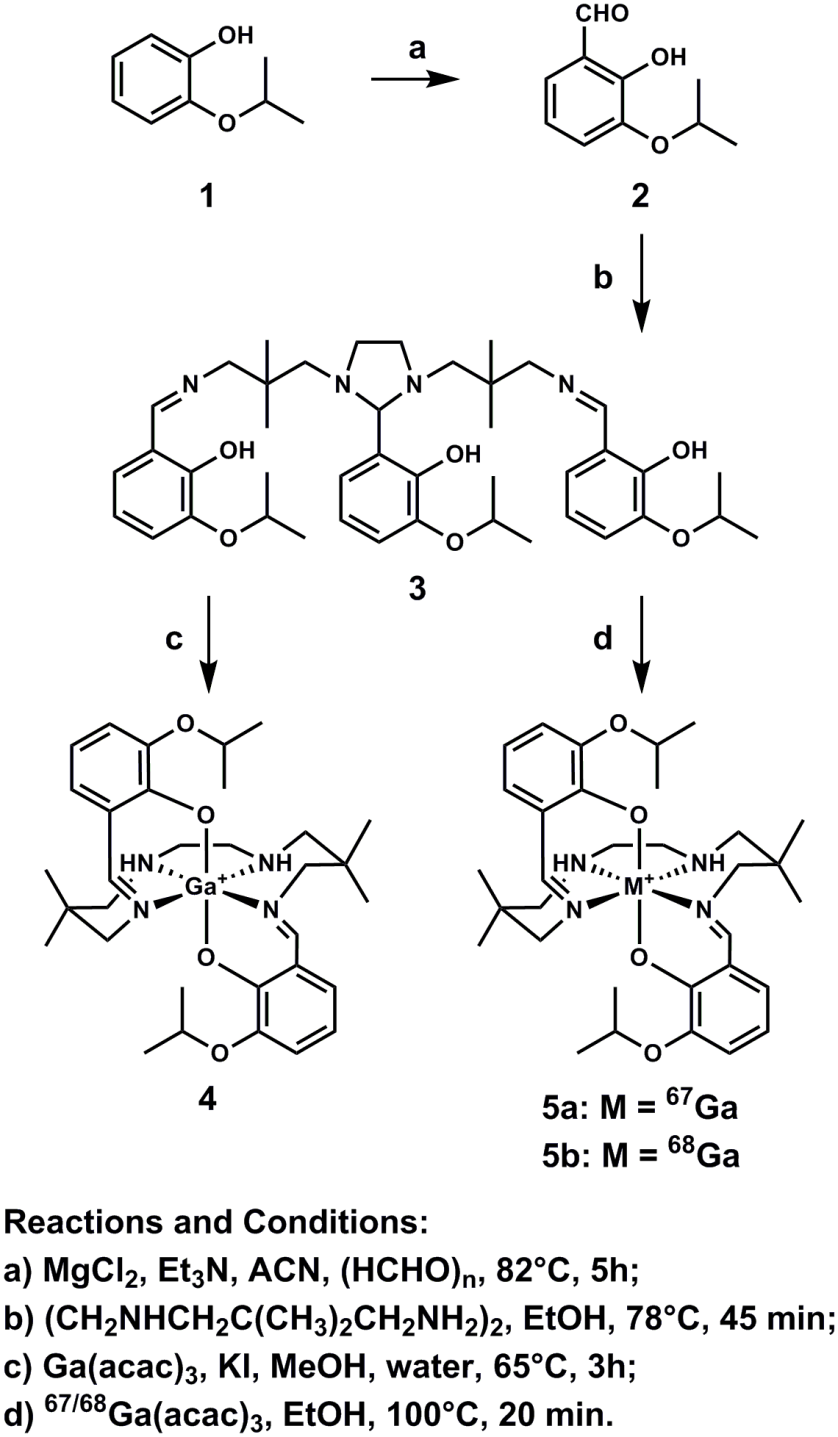
Scheme for chemical synthesis of gallium(III) complexes (4, 5a, and 5b).

**Figure 2:**
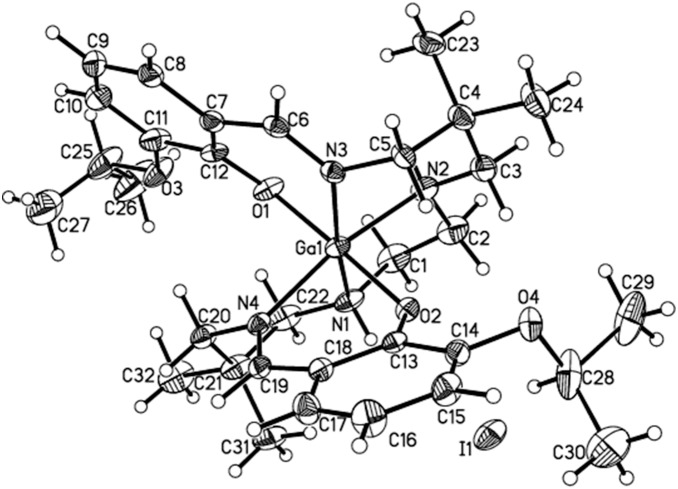
Projection view of the cationic gallium(III) complex Ga-[3-isopropoxy-ENBDMPI]^+^ (4), with the iodide (I^−^) counteranion, showing the crystallographic numbering scheme.

Herein we report and characterize gallium(III)-(bis(3-isopropoxy-2-phenolate-benzylidene)-N,N’-bis(2,2-dimethyl-3-amino-propyl)ethylenediamine) (Ga-[3-isopropoxy-ENBDMPI]^+^), **4**, a novel hydrophobic monocationic Ga(III)-complex. Isolated as pale yellow crystals in 30% yield, **4** was synthesized from the condensation of a linear tetraamine and 2-hydroxy-3-isopropoxy-benzaldehyde (**2**, obtained via orthoformulation of 2-isopropoxy phenol **1** using MgCl_2_ and paraformaldehyde) followed by a ligand-exchange reaction involving **3** and Ga(acetylacetonate)_3_ in methanol as shown in **Fig. 1** [15], [30]. The molecular structure of the isopropoxy pendant Ga(III)-complex determined by x-ray diffraction (**Fig. 2**) demonstrates pseudo-octahedral geometry, wherein the central metal is surrounded by two secondary amine nitrogen atoms of the hydrocarbon backbone, two aldimino nitrogen atoms in the equatorial plane, and two axial phenolate oxygen atoms. Furthermore, upon encompassing the central metal atom, the N_4_O_2_ donor core of the ligand results in formation of four six-membered rings and one five-membered ring involving participation of the Ga1-N2-C2-C1-N1 atoms. Additionally, Ga-O distances average 1.9279 Å, while Ga-N distances average 2.0719 Å, consistent with formation of covalent and coordinate bonds, respectively. While trans bond angles O1-Ga-O2, N1-Ga-N3, N2-Ga-N4 average 171.6°, the cis angles centered around O-Ga-N average 90.1°, indicating minimal distortion from ideal octahedral geometry for complex **4**. Finally, ^1^H NMR and proton-decoupled ^13^C NMR spectra of **4** recorded in DMSO-*d*_6_ demonstrate that the aromatic rings remain chemically equivalent upon coordination of the donor core of ligand **3**, thus supporting the existence of a 2-fold symmetry for the complex in solution. These data are in accord with other metal complexes of related ligands [14], [15], [17], [20]–[22], [24], [31], [32], both in solution and solid state.

For assessment of the Ga(III)-complex *in cellulo* and *in vivo*, radiolabeled ^67^Ga- and ^68^Ga-complexes (**5a** and **5b**) were prepared with radioactive Ga(acetylacetonate)_3_ and ligand **3** in ethanol at 100°C for 40 min (**Fig. 1** **)** [20], [22], [24]. Synthesis and purification of the radiopharmaceutical can be accomplished in <1 hr, an interval of significant practical value for synthesis of PET agents with ^68^Ga. Nonetheless, to perform cell tracer assays and pharmacokinetic studies in mice and rats, ^67^Ga-complexes were generated to exploit the longer half-life of ^67^Ga (t_½_  = 78.2 hr), thereby allowing cellular analysis and *in vivo* studies with the same HPLC purified fraction (C-18 column; ethanol/saline gradient; R_t_ = 27.0 min). Formation of radio-complexes **5a** and **5b** was also monitored via radio-TLC (methanol/saline (90/10); R_f_ = 0.23; radiochemical purity >95%; radiochemical yield 60%).

Mechanistic studies of cationic metal-complexes useful for MPI have revealed that net retention of tracers in heart tissue is determined by the opposing action of two biochemical processes [20], [33]. First, effective metal-complexes are hydrophobic monocations, permeating passively into living cells and concentrating within the mitochondrial inner matrix in response to the driving forces of electronegative plasma membrane and mitochondrial transmembrane potentials [29]. This is opposed by the action of ATP-binding-cassette (ABC) membrane transporters, such as the multidrug resistance (MDR) P-glycoprotein (Pgp; *ABCB1*) and MRP1 (*ABCC1*), which transport hydrophobic cationic metal-complexes out of cells [24], [33], [34]. Thus, cardiomyocytes, rich in mitochondria and lacking Pgp, sequester metal-complexes for prolonged periods, while hepatocytes, which express Pgp along their canalicular surface, rapidly excrete metal-complexes into the bile and intestines. In principle, if translated *in vivo*, these properties would produce rapid hepatocellular clearance of the agent, thereby minimizing the impact of emissions arising from the liver that could mis-register into the inferior wall of the myocardium during imaging. Consideration of these factors provided a platform for designing hydrophobic monocationic gallium(III)-complexes that were simultaneously membrane potential-responsive and avidly transported by Pgp [20]. In addition, structure-activity analysis of related metal complexes suggested that ester and ether moieties may confer Pgp recognition properties [35]–[37].

We first demonstrated the membrane potential-responsive uptake of ^67^Ga(III)-complex **5a** in rat H9c2 cardiomyoblasts. Complex **5a** accumulated into H9c2 cells to high levels (745±112 fmol (mg P)^−1^ (nM_0_)^−1^). Depolarizing cardiac sarcolemmal and mitochondrial membrane potentials with 130 mM K^+^/20 mM Cl^−^ buffer containing the potassium ionophore valinomycin (1 µg/mL) eliminates the inward driving force for charged complexes [33], and decreased **5a** content to 155±17 fmol (mg P)^−1^ (nM_0_)^−1^, <20% of control, indicating a significant contribution from electronegative membrane potentials driving cell uptake of **5a**.

**Figure 3:**
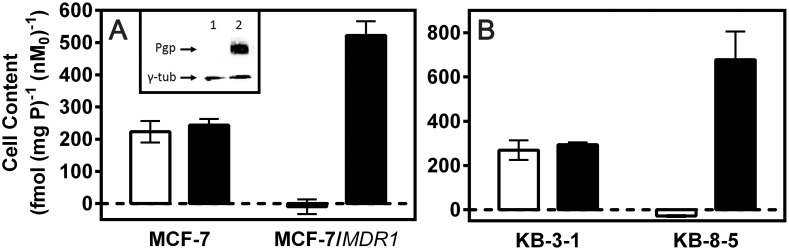
Characterization of ^67^Ga-labeled 5a *in cellulo*. **A.** Cellular accumulation of **5a** in human MCF-7 cells (Pgp-) and stably transfected MCF-7/*MDR1* (Pgp+) cells. *Inset*: Immunodetectable levels of Pgp in plasma membrane preparations using mAb C219; MCF-7 cells (lane 1) and MCF-7/*MDR1* cells (lane 2). Arrows demarcate Pgp (170 kDa) and γ-tubulin (γ-t) loading control. **B.** Cellular accumulation of **5a** in KB-3–1 cells (Pgp-) and KB-8–5 (Pgp+). Shown are net uptakes at 90 min (fmol (mg protein)^−1^ (nM_0_)^−1^) using control buffer in the absence (open column) or presence (solid column) of the MDR modulator LY335979 (1 µM). Each column represents the mean of 4 determinations; bar denotes ±SEM.

Next, to determine whether Pgp affected cellular accumulation of **5a**, parental MCF-7 cells and a subclone stably transfected with *MDR1* Pgp were used for transport studies. Control MCF-7 cells lack immunodetectable Pgp, whereas MCF-7/*MDR1* cells express biologically relevant Pgp levels by Western blot analysis [26], [38] (**Fig. 3a**). Accumulation of ^67^Ga-complex **5a** was high in parental MCF-7 cells and indistinguishable from zero in MCF-7/*MDR1* cells (**Fig. 3a**). Furthermore, treatment with the potent MDR modulator LY335979 at a concentration known to maximally block Pgp (1 µM) [38] fully reversed the accumulation defect of **5a** in MCF-7/*MDR1* cells, consistent with inhibition of Pgp-mediated efflux of **5a**. Importantly, LY335979 (1 µM) did not significantly impact uptake of **5a** in MCF-7 cells. Furthermore, drug sensitive KB-3-1 cells, which lack immunodetectable Pgp, accumulated **5a** to high levels, while drug-resistant KB-8-5 cells, which express modest Pgp levels by Western blot analysis [26], [38], showed no accumulation. As anticipated, LY335979 enhanced cellular accumulation of **5a** in the MDR KB-8-5 cells, but not drug-sensitive KB-3-1 cells (**Fig. 3b**).

**Figure 4:**
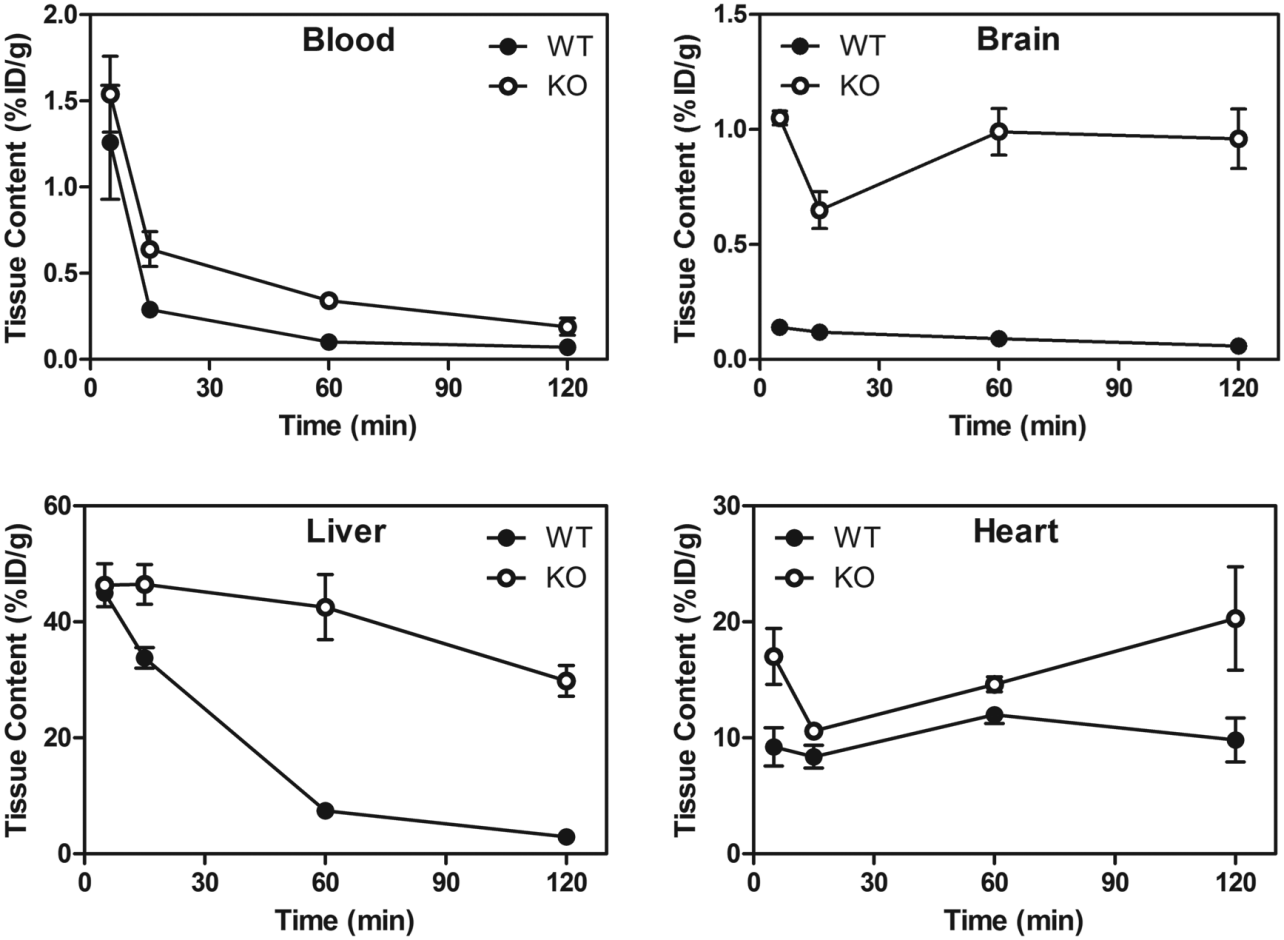
Pharmacokinetic data of 5a in WT and *mdr1a/1b^(−/−)^* mice (n = 3). In WT mice, **5a** clears from the liver and does not penetrate significantly into the brain, but shows high and stable retention in heart tissue (a Pgp negative organ) in both WT and *mdr1a/1b^(−/−)^* mice. Values represent the mean of 3 determinations; bar denotes ±SEM.

**Figure 5:**
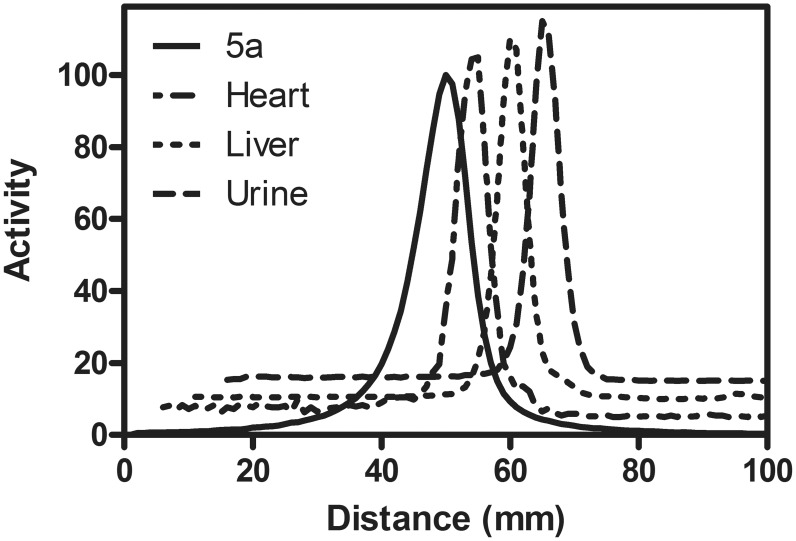
Representative metabolite analysis of ^67^Ga-labeled 5a at 60 min post injection into a Sprague-Dawley rat (a time point compatible with ^68^Ga imaging). Peaks are offset for clarity. Note the presence of a parental compound in all tissues.

We then determined whether Pgp impacted the pharmacokinetics of **5a** in mice *in vivo*. Mice possess two drug-transporting isoforms of Pgp (*mdr1a* and *mdr1b*) [39]. Both drug-transporting isoforms are expressed along the biliary cannalicular surface of hepatocytes wherein the protein functions to secrete substrates into the bile, while the *mdr1a* isoform is expressed on the luminal surface of capillary endothelial cells of the brain wherein Pgp imparts important exclusion functionality to the blood-brain barrier [40]. Following intravenous injection in wild type (WT) mice, **5a** demonstrated fast clearance from the blood pool (%ID/g = 1.26±0.33 (5 min), 0.10±0.01 (60 min)). By contrast, blood retention 60 minutes post intravenous injection of **5a** in gene-deleted *mdr1a/1b^(−/−)^* mice was 0.34±0.03%ID/g, 3.4-fold greater than WT control (**Fig. 4**), a result likely reflecting the expression of Pgp in WT leukocytes [41]. Importantly, the heart, a Pgp negative tissue, showed robust uptake and retention in both WT (%ID/g = 12.0±0.7) and *mdr1a/1b^(−/−)^* (%ID/g = 14.6±0.6) mice 60 min post injection of the radiotracer (**Fig. 4** **;** **Tables 1** **and** **2**). In liver, *initial* accumulation of **5a** was comparable between *mdr1a/1b^(−/−)^* and WT mice 5 min post injection of the tracer (**Fig. 4**). However, liver clearance at 60 min was markedly delayed in *mdr1a/1b^−/−^* mice, showing 6-fold higher retention compared with WT mice, consistent with Pgp-mediated biliary secretion of the tracer. Relative to WT mice, *mdr1a/1b^(−/−)^* mice showed an 11-fold higher retention of the ^67^Ga-complex in brain parenchyma 60 minutes post injection (**Fig. 4** **,** **Tables 1** **and** **2**). In WT mice, heart/blood and heart/liver ratios of **5a** at 60 min post injection were 130 and 1.6, respectively (**Table 3**). Additional pharmacokinetic studies were conducted in WT Sprague-Dawley male rats (n = 3) (**Table S7**). Pharmacokinetic profiles in rats were found to be similar to that of WT mice, indicative of Pgp-mediated excretion pathways. Importantly,^ 67^Ga-complex **5a** showed high extraction and sustained retention in the heart with rapid clearance from the blood pool and liver, yielding heart/blood ratios of 8.4 (5 min), 50 (60 min) and 139 (120 min), and heart/liver ratios of 0.6 (5 min), 4.2 (60 min), and 7.8 (120 min), respectively (**Table S8**). Transferrin, a serum protein, has two iron(III) binding sites with high affinity for Ga(III). Therefore, dechelation of the radionuclide (Ga^+3^) from **5a** could exchange with the iron of transferrin, leading to retention of activity within the blood pool, and tissues. For analysis, radioHPLC allows detection of only mobile species, while radioTLC enables assessment of both mobile and immobile radiometric species. Therefore, we assessed samples of tissue extracts using radioTLC for preliminary evaluation of metabolites. Importantly, radioTLC analysis of extracts from heart, liver, blood and urine 30, 60, and 90 min post tail-vein injection of **5a** in rats showed only the existence of parental compound (**Fig.** **5**), thus demonstrating the high stability of the radiopharmaceutical *in vivo*. These results are consistent with facile clearance of **5a** from the blood pool and liver tissue of rats.

**Table 1:** Biodistribution Data (%ID/g) for ^67^Ga-Complex **5a** in WT mice (n = 3).

Time (min) P.I.	5		15		60		120	
%ID/g	Mean	SEM	Mean	SEM	Mean	SEM	Mean	SEM
blood	1.26	0.33	0.29	0.04	0.10	0.01	0.07	0.01
liver	44.95	1.24	33.80	1.80	7.44	0.44	2.90	0.24
kidneys	81.04	17.46	83.46	10.00	93.35	14.49	67.91	5.59
heart	9.21	1.64	8.37	0.98	11.98	0.74	9.81	1.90
brain	0.14	0.01	0.12	0.02	0.09	0.01	0.06	0.01

**Table 2:** Biodistribution Data (%ID/g) for ^67^Ga-Complex **5a** in *mdr1a/1b* ^(−/−)^ mice (n = 3).

Time (min) P.I.	5		15		60		120	
%ID/g	Mean	SEM	Mean	SEM	Mean	SEM	Mean	SEM
blood	1.54	0.22	0.64	0.10	0.34	0.03	0.19	0.05
liver	46.34	3.71	46.45	3.42	42.54	5.61	29.82	2.66
kidneys	86.12	4.33	84.19	7.62	95.60	10.38	115.23	10.09
heart	17.02	2.42	10.59	0.58	14.61	0.64	20.29	4.45
brain	1.05	0.03	0.65	0.08	0.99	0.10	0.96	0.13

**Table 3:** Heart to Tissue Ratio of ^67^Ga-Complex **5a** in WT mice (n = 3).

Time (min) P.I.	5		60		120	
%ID/g	Mean	SEM	Mean	SEM	Mean	SEM
Heart/Blood	7.8	1.1	127	18	147	25
Heart/Liver	0.21	0.04	1.6	0.12	3.5	0.88

**Figure 6:**
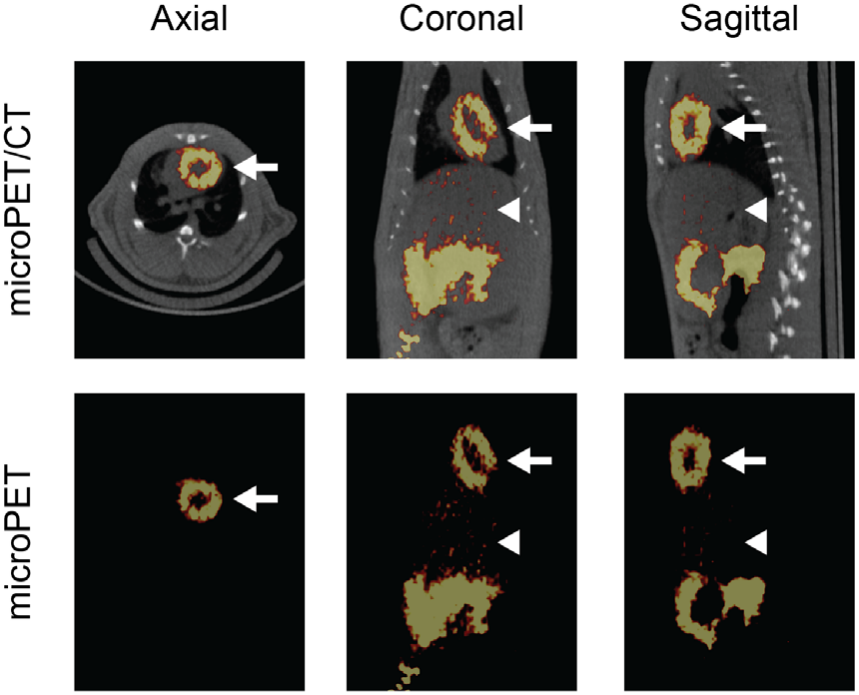
Representative MicroPET/CT Images. Sprague-Dawley rats (n = 3) were injected intravenously with HPLC-purified ^68^Ga-labeled **5b** (600 µCi) and images were acquired 60 min post injection. **Top Panel**, microPET images co-registered with CT; **Lower Panel**, microPET images alone. Note retention of **5b** in the heart (white arrow) and rapid clearance of the tracer from the liver (white arrow head) into the intestines, properties ideal for a myocardial perfusion imaging agent.

While screening candidate radiopharmaceuticals *in cellulo* identifies mechanism-based leads, many characteristics vital for development *in vivo* relate to serum protein binding, pharmacokinetics, tissue retention, and overall signal-to-noise that are difficult to predict and may adversely influence image quality. Indeed, several promising synthetic complexes of Ga(III) have been previously reported, but failed to generate high quality images *in vivo* [22], [23], [42]. Thus, to begin to characterize the properties of **5b** as a MPI *in vivo*, microPET/CT imaging was performed on rats following i.v. injection of HPLC-purified complex. Analysis of the images revealed sustained retention of **5b** in heart tissue and rapid clearance from the liver (**Fig. 6**). The entire left ventricular wall and septum were clearly visualized on PET images with high diagnostic quality 60 min post injection and there was evidence of right ventricular wall visualization. Additionally, small animal SPECT/CT images with ^67^Ga-complex **5a** 60 min post injection were similar to the PET images (data not shown). SUV values derived from the SPECT/CT study showed a heart/liver ratio of 3.3, a value with excellent prospects for further development.

## Conclusions

A novel ^68^Ga(III)-complex identified by mechanism-based cell screening holds substantial promise as a PET MPI radiopharmaceutical. While several agents in this class have been synthesized and reported, no prior agent has shown the favorable MPI properties of **5b** in pre-clinical models *in vivo*. This includes both high contrast perfusion images of the heart combined with rapid clearance from the liver, accomplished by a rational design targeting Pgp transporter-mediated excretion. The design of **5b** includes aromatic scaffolds substituted with alkoxy groups, a characteristic structural feature among several cationic metal-complexes recognized as Pgp substrates [20], [36], [37], [43]. Thus, incorporation of an isopropoxy functionality into the 3-position of the aromatic ring, which may provide resonance stabilization [44], has significant structural consequences and indicates that properly designed pendant moieties are capable of promoting high myocardial uptake and rapid liver clearance of radioactive Ga(III)-complexes. Because ^68^Ga is generator-produced on-site, the ready formulation of **5b** provides a promising alternative for PET MPI, enabling point-of-care radiopharmaceutical distribution logistics temporally responsive to the urgent care needs of individual patients. Given the biological targeting properties of **5b**, the complex also may enable PET imaging of MDR in tumors [24], [33], [45], Pgp activity at the blood brain barrier in neurodegenerative diseases, such as Alzheimer’s disease and Parkinson disease [43], [46], as well as detection of mitochondrial myopathies *in vivo* [47].

## Supporting Information

Figure S1 **Projection view (50% thermal ellipsoids) of cationic gallium(III) complex Ga-[3-isopropoxy-ENBDMPI]^+^ (4) with iodide (I^−^) as a counter anion showing the crystallographic numbering scheme.** Solvent and isopropyl disorders are omitted for better presentation.
(TIF)

Figure S2 **Projection view (50% thermal ellipsoids) of cationic gallium(III) complex Ga-[3-isopropoxy-ENBDMPI]^+^ (4) with the iodide (I^−^) as a counter anion showing the crystallographic numbering scheme.** While solvent and isopropyl disorders are included, the hydrogen atoms are excluded for a clear presentation.
(TIF)

Table S1 **Crystal data and structure refinement for [ENBDMP-3-isopropoxy-PI-Ga]^+^ I^−^ (4).**
(DOCX)

Table S2 **Atomic coordinates (x 10^4^) and equivalent isotropic displacement parameters (Å^2×^10^3^) for [ENBDMP-3-isopropoxy-PI-Ga]^+^ I^−^ (4).** U(eq) is defined as one third of the trace of the orthogonalized U^ij^ tensor.
(DOCX)

Table S3 **Bond lengths [Å] and angles [°] for [ENBDMP-3-isopropoxy-PI-Ga]^+^ I^−^ (4).**
(DOCX)

Table S4 **Anisotropic displacement parameters (Å^2×^10^3^) for [ENBDMP-3-isopropoxy-PI-Ga]^+^ I^−^ (4).** The anisotropic displacement factor exponent takes the form: −2π^2^[h^2^ a*^2^ U^11^+… +2 h k a* b* U^12^].
(DOCX)

Table S5 **Hydrogen coordinates (x 10^4^) and isotropic displacement parameters (Å^2×^10^3^) for [ENBDMP-3-isopropoxy-PI-Ga]^+^ I^−^ (4).**
(DOCX)

Table S6 **Torsion angles [°] for [ENBDMP-3-isopropoxy-PI-Ga]^+^ I^−^ (4).**
(DOCX)

Table S7 **Biodistribution Data (%ID/g) for ^67^Ga-Complex 5a in Sprague Dawley rats (n = 3).**
(DOCX)

Table S8 **Heart to Tissue Ratio of ^67^Ga-Complex 5a in Sprague-Dawley rats (n = 3).**
(DOCX)
