# Supplementary figures and images for: A Generator-Produced Gallium-68 Radiopharmaceutical for PET Imaging of Myocardial Perfusion
Source: PLoS One. 2014 Oct 29;9(10):e109361. doi: 10.1371/journal.pone.0109361 (PMC4212944; doi:10.1371/journal.pone.0109361)

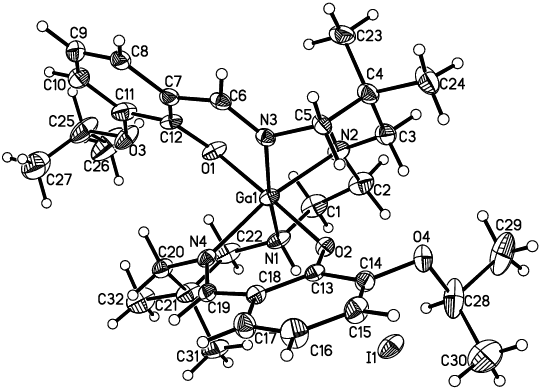

Supplement: Figure S1 — Projection view (50% thermal ellipsoids) of cationic gallium(III) complex Ga-[3-isopropoxy-ENBDMPI]+ (4) with iodide (I−) as a counter anion showing the crystallographic numbering scheme. Solvent and isopropyl disorders are omitted for better presentation. (TIF) [file pone.0109361.s001.tif]

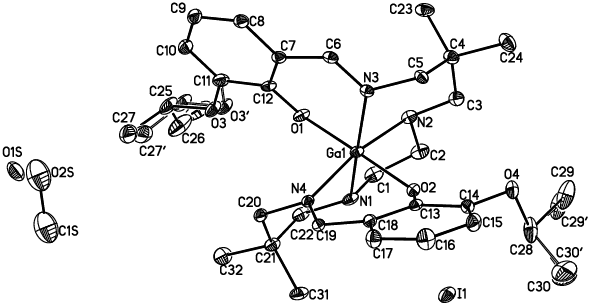

Supplement: Figure S2 — Projection view (50% thermal ellipsoids) of cationic gallium(III) complex Ga-[3-isopropoxy-ENBDMPI]+ (4) with the iodide (I−) as a counter anion showing the crystallographic numbering scheme. While solvent and isopropyl disorders are included, the hydrogen atoms are excluded for a clear presentation. (TIF) [file pone.0109361.s002.tif]
