# Supplementary material for: A Generator-Produced Gallium-68 Radiopharmaceutical for PET Imaging of Myocardial Perfusion
Source: PLoS One. 2014 Oct 29;9(10):e109361. doi: 10.1371/journal.pone.0109361 (PMC4212944; doi:10.1371/journal.pone.0109361)
Supplement: Table S1 — Crystal data and structure refinement for [ENBDMP-3-isopropoxy-PI-Ga]+ I− (4). (DOCX) [file pone.0109361.s003.docx]

**Table S1.** Crystal data and structure refinement for [ENBDMP-3-isopropoxy-PI-Ga]^+^ I^-^ **(4)**.

Identification code v6310/lt/JOT-1-213

Empirical formula C_32.25_ H_50.5_ Ga I N_4_ O_5.25_

Formula weight 774.76

Temperature 100(2) K

Wavelength 0.71073 Å

Crystal system Orthorhombic

Space group Pbca

Unit cell dimensions a = 22.6349(11) Å α = 90°.

b = 13.5631(6) Å β = 90°.

c = 23.4581(11) Å γ = 90°.

Volume 7201.6(6) Å^3^

Z 8

Density (calculated) 1.429 Mg/m^3^

Absorption coefficient 1.664 mm^-1^

F(000) 3183

Crystal size 0.36 x 0.33 x 0.21 mm^3^

Theta range for data collection 1.74 to 27.22°.

Index ranges -28≤h≤28, -17≤k≤17, -30≤l≤30

Reflections collected 205315

Independent reflections 7980 [R(int) = 0.0395]

Completeness to theta = 25.00° 99.9 %

Absorption correction Semi-empirical from equivalents

Max. and min. transmission 0.7255 and 0.5864

Refinement method Full-matrix least-squares on F^2^

Data / restraints / parameters 7980 / 55 / 453

Goodness-of-fit on F2 1.084

Final R indices [I>2sigma(I)] R1 = 0.0288, wR2 = 0.0686

R indices (all data) R1 = 0.0361, wR2 = 0.0752

Largest diff. peak and hole 1.384 and -1.019 e.Å^-3^
