# Supplementary material for: A Generator-Produced Gallium-68 Radiopharmaceutical for PET Imaging of Myocardial Perfusion
Source: PLoS One. 2014 Oct 29;9(10):e109361. doi: 10.1371/journal.pone.0109361 (PMC4212944; doi:10.1371/journal.pone.0109361)
Supplement: Table S2 — Atomic coordinates (x 104) and equivalent isotropic displacement parameters (Å2×103) for [ENBDMP-3-isopropoxy-PI-Ga]+ I− (4). U(eq) is defined as one third of the trace of the orthogonalized Uij tensor. (DOCX) [file pone.0109361.s004.docx]

**Table S2.** Atomic coordinates (x 10^4^) and equivalent isotropic displacement parameters (Å^2^ x 10^3^) for [ENBDMP-3-isopropoxy-PI-Ga]^+^ I^-^ **(4)**. U(eq) is defined as one third of the trace of the orthogonalized U^ij^ tensor.

______________________________________________________________________

x y z U(eq)

______________________________________________________________________

I(1) 10530(1) 2546(1) 4297(1) 36(1)

Ga(1) 9952(1) 2374(1) 6385(1) 17(1)

O(1) 9200(1) 2674(1) 6730(1) 24(1)

O(2) 10703(1) 2028(1) 6052(1) 18(1)

O(4) 11910(1) 2150(1) 6096(1) 26(1)

N(1) 9544(1) 2607(1) 5593(1) 23(1)

N(2) 10197(1) 3848(1) 6296(1) 23(1)

N(3) 10313(1) 2432(1) 7186(1) 17(1)

N(4) 9726(1) 916(1) 6295(1) 18(1)

C(1) 9521(1) 3684(2) 5501(1) 31(1)

C(2) 10097(1) 4124(2) 5692(1) 30(1)

C(3) 10806(1) 4100(2) 6475(1) 25(1)

C(4) 10954(1) 3903(2) 7099(1) 24(1)

C(5) 10920(1) 2796(1) 7247(1) 19(1)

C(6) 10019(1) 2313(1) 7651(1) 18(1)

C(7) 9390(1) 2171(1) 7693(1) 20(1)

C(8) 9158(1) 1928(2) 8233(1) 24(1)

C(9) 8563(1) 1866(2) 8314(1) 28(1)

C(10) 8180(1) 2041(2) 7859(1) 30(1)

C(11) 8394(1) 2282(2) 7329(1) 29(1)

C(12) 9014(1) 2377(2) 7232(1) 21(1)

C(13) 11003(1) 1243(1) 6210(1) 17(1)

C(14) 11629(1) 1276(2) 6226(1) 21(1)

C(15) 11953(1) 475(2) 6398(1) 26(1)

C(16) 11681(1) -406(2) 6550(1) 31(1)

C(17) 11077(1) -471(2) 6523(1) 26(1)

C(18) 10735(1) 345(2) 6363(1) 18(1)

C(19) 10104(1) 213(2) 6309(1) 18(1)

C(20) 9113(1) 665(2) 6157(1) 23(1)

C(21) 8936(1) 1034(2) 5560(1) 26(1)

C(22) 8953(1) 2154(2) 5515(1) 28(1)

C(23) 10558(1) 4489(2) 7502(1) 31(1)

C(24) 11597(1) 4219(2) 7184(1) 35(1)

C(28) 11948(1) 2332(2) 5484(1) 42(1)

C(29) 12138(9) 3399(6) 5521(9) 52(2)

C(30) 12420(30) 1750(60) 5180(20) 56(3)

C(29') 12017(6) 3419(3) 5362(7) 52(2)

C(30') 12447(15) 1700(30) 5263(11) 56(3)

C(31) 9320(1) 559(2) 5104(1) 32(1)

C(32) 8294(1) 707(2) 5471(1) 40(1)

O(3) 8051(2) 2414(6) 6872(2) 32(1)

C(25) 7422(2) 2329(7) 6927(2) 41(2)

C(26) 7179(3) 2871(9) 6421(3) 65(2)

C(27) 7239(3) 1251(8) 6934(3) 60(2)

O(3') 8093(6) 2698(14) 6799(8) 32(1)

C(25') 7458(7) 2702(19) 6829(8) 41(2)

C(26') 7239(11) 3440(30) 6420(9) 66(7)

C(27') 7210(10) 1680(20) 6761(15) 66(6)

O(1S) 5645(1) 385(2) 6646(1) 53(1)

O(2S) 6604(8) -452(11) 6996(7) 89(6)

C(1S) 6580(12) -792(16) 6390(8) 89(6)

_________________________________________________________________
