# Supplementary material for: A Generator-Produced Gallium-68 Radiopharmaceutical for PET Imaging of Myocardial Perfusion
Source: PLoS One. 2014 Oct 29;9(10):e109361. doi: 10.1371/journal.pone.0109361 (PMC4212944; doi:10.1371/journal.pone.0109361)
Supplement: Table S3 — Bond lengths [Å] and angles [°] for [ENBDMP-3-isopropoxy-PI-Ga]+ I− (4). (DOCX) [file pone.0109361.s005.docx]

**Table S3.** Bond lengths [Å] and angles [°] for [ENBDMP-3-isopropoxy-PI-Ga]^+^ I^-^ **(4)**.

_____________________________________________________

Ga(1)-O(2) 1.9277(14)

Ga(1)-O(1) 1.9287(15)

Ga(1)-N(3) 2.0506(17)

Ga(1)-N(4) 2.0528(17)

Ga(1)-N(2) 2.0850(18)

Ga(1)-N(1) 2.0985(18)

O(1)-C(12) 1.315(3)

O(2)-C(13) 1.316(2)

O(4)-C(14) 1.379(3)

O(4)-C(28) 1.460(3)

N(1)-C(1) 1.478(3)

N(1)-C(22) 1.482(3)

N(1)-H(1) 0.86(3)

N(2)-C(3) 1.481(3)

N(2)-C(2) 1.483(3)

N(2)-H(2) 0.82(3)

N(3)-C(6) 1.286(3)

N(3)-C(5) 1.467(3)

N(4)-C(19) 1.282(3)

N(4)-C(20) 1.464(3)

C(1)-C(2) 1.502(4)

C(1)-H(1A) 0.9900

C(1)-H(1B) 0.9900

C(2)-H(2A) 0.9900

C(2)-H(2B) 0.9900

C(3)-C(4) 1.525(3)

C(3)-H(3A) 0.9900

C(3)-H(3B) 0.9900

C(4)-C(23) 1.527(3)

C(4)-C(24) 1.529(3)

C(4)-C(5) 1.542(3)

C(5)-H(5A) 0.9900

C(5)-H(5B) 0.9900

C(6)-C(7) 1.439(3)

C(6)-H(6) 0.9500

C(7)-C(12) 1.405(3)

C(7)-C(8) 1.411(3)

C(8)-C(9) 1.361(3)

C(8)-H(8) 0.9500

C(9)-C(10) 1.396(3)

C(9)-H(9) 0.9500

C(10)-C(11) 1.375(3)

C(10)-H(10) 0.9500

C(11)-O(3) 1.336(5)

C(11)-C(12) 1.426(3)

C(11)-O(3') 1.524(17)

C(13)-C(18) 1.408(3)

C(13)-C(14) 1.418(3)

C(14)-C(15) 1.372(3)

C(15)-C(16) 1.391(3)

C(15)-H(15) 0.9500

C(16)-C(17) 1.372(3)

C(16)-H(16) 0.9500

C(17)-C(18) 1.402(3)

C(17)-H(17) 0.9500

C(18)-C(19) 1.445(3)

C(19)-H(19) 0.9500

C(20)-C(21) 1.540(3)

C(20)-H(20A) 0.9900

C(20)-H(20B) 0.9900

C(21)-C(31) 1.522(3)

C(21)-C(22) 1.524(3)

C(21)-C(32) 1.534(3)

C(22)-H(22A) 0.9900

C(22)-H(22B) 0.9900

C(23)-H(23A) 0.9800

C(23)-H(23B) 0.9800

C(23)-H(23C) 0.9800

C(24)-H(24A) 0.9800

C(24)-H(24B) 0.9800

C(24)-H(24C) 0.9800

C(28)-C(30) 1.509(5)

C(28)-C(30') 1.509(4)

C(28)-C(29') 1.510(4)

C(28)-C(29) 1.513(5)

C(28)-H(28) 1.0000

C(29)-H(29A) 0.9800

C(29)-H(29B) 0.9800

C(29)-H(29C) 0.9800

C(30)-H(30A) 0.9800

C(30)-H(30B) 0.9800

C(30)-H(30C) 0.9800

C(29')-H(29D) 0.9800

C(29')-H(29E) 0.9800

C(29')-H(29F) 0.9800

C(30')-H(30D) 0.9800

C(30')-H(30E) 0.9800

C(30')-H(30F) 0.9800

C(31)-H(31A) 0.9800

C(31)-H(31B) 0.9800

C(31)-H(31C) 0.9800

C(32)-H(32A) 0.9800

C(32)-H(32B) 0.9800

C(32)-H(32C) 0.9800

O(3)-C(25) 1.436(4)

C(25)-C(26) 1.500(6)

C(25)-C(27) 1.520(7)

C(25)-H(25) 1.0000

C(26)-H(26A) 0.9800

C(26)-H(26B) 0.9800

C(26)-H(26C) 0.9800

C(27)-H(27A) 0.9800

C(27)-H(27B) 0.9800

C(27)-H(27C) 0.9800

O(3')-C(25') 1.440(14)

C(25')-C(26') 1.473(15)

C(25')-C(27') 1.507(16)

C(25')-H(25') 1.0000

C(26')-H(26D) 0.9800

C(26')-H(26E) 0.9800

C(26')-H(26F) 0.9800

C(27')-H(27D) 0.9800

C(27')-H(27E) 0.9800

C(27')-H(27F) 0.9800

O(1S)-H(1S) 0.9441

O(2S)-C(1S) 1.496(5)

O(2)-Ga(1)-O(1) 178.01(6)

O(2)-Ga(1)-N(3) 91.68(6)

O(1)-Ga(1)-N(3) 87.66(7)

O(2)-Ga(1)-N(4) 86.84(6)

O(1)-Ga(1)-N(4) 91.48(7)

N(3)-Ga(1)-N(4) 103.34(6)

O(2)-Ga(1)-N(2) 87.59(7)

O(1)-Ga(1)-N(2) 94.25(7)

N(3)-Ga(1)-N(2) 87.05(7)

N(4)-Ga(1)-N(2) 168.34(7)

O(2)-Ga(1)-N(1) 93.82(7)

O(1)-Ga(1)-N(1) 87.15(7)

N(3)-Ga(1)-N(1) 168.71(7)

N(4)-Ga(1)-N(1) 86.81(7)

N(2)-Ga(1)-N(1) 83.34(7)

C(12)-O(1)-Ga(1) 126.37(13)

C(13)-O(2)-Ga(1) 122.52(12)

C(14)-O(4)-C(28) 112.97(17)

C(1)-N(1)-C(22) 111.06(18)

C(1)-N(1)-Ga(1) 107.11(14)

C(22)-N(1)-Ga(1) 116.38(14)

C(1)-N(1)-H(1) 106.3(18)

C(22)-N(1)-H(1) 106.1(18)

Ga(1)-N(1)-H(1) 109.5(19)

C(3)-N(2)-C(2) 110.77(18)

C(3)-N(2)-Ga(1) 116.14(13)

C(2)-N(2)-Ga(1) 107.31(14)

C(3)-N(2)-H(2) 108.4(18)

C(2)-N(2)-H(2) 105.4(18)

Ga(1)-N(2)-H(2) 108.3(19)

C(6)-N(3)-C(5) 116.40(17)

C(6)-N(3)-Ga(1) 124.45(14)

C(5)-N(3)-Ga(1) 118.31(13)

C(19)-N(4)-C(20) 117.72(18)

C(19)-N(4)-Ga(1) 123.14(14)

C(20)-N(4)-Ga(1) 118.89(13)

N(1)-C(1)-C(2) 108.57(18)

N(1)-C(1)-H(1A) 110.0

C(2)-C(1)-H(1A) 110.0

N(1)-C(1)-H(1B) 110.0

C(2)-C(1)-H(1B) 110.0

H(1A)-C(1)-H(1B) 108.4

N(2)-C(2)-C(1) 108.51(19)

N(2)-C(2)-H(2A) 110.0

C(1)-C(2)-H(2A) 110.0

N(2)-C(2)-H(2B) 110.0

C(1)-C(2)-H(2B) 110.0

H(2A)-C(2)-H(2B) 108.4

N(2)-C(3)-C(4) 115.81(18)

N(2)-C(3)-H(3A) 108.3

C(4)-C(3)-H(3A) 108.3

N(2)-C(3)-H(3B) 108.3

C(4)-C(3)-H(3B) 108.3

H(3A)-C(3)-H(3B) 107.4

C(3)-C(4)-C(23) 112.01(19)

C(3)-C(4)-C(24) 106.50(18)

C(23)-C(4)-C(24) 109.35(19)

C(3)-C(4)-C(5) 112.01(17)

C(23)-C(4)-C(5) 109.79(18)

C(24)-C(4)-C(5) 106.97(18)

N(3)-C(5)-C(4) 110.67(17)

N(3)-C(5)-H(5A) 109.5

C(4)-C(5)-H(5A) 109.5

N(3)-C(5)-H(5B) 109.5

C(4)-C(5)-H(5B) 109.5

H(5A)-C(5)-H(5B) 108.1

N(3)-C(6)-C(7) 125.89(19)

N(3)-C(6)-H(6) 117.1

C(7)-C(6)-H(6) 117.1

C(12)-C(7)-C(8) 120.7(2)

C(12)-C(7)-C(6) 121.31(19)

C(8)-C(7)-C(6) 117.55(19)

C(9)-C(8)-C(7) 120.5(2)

C(9)-C(8)-H(8) 119.7

C(7)-C(8)-H(8) 119.7

C(8)-C(9)-C(10) 119.9(2)

C(8)-C(9)-H(9) 120.1

C(10)-C(9)-H(9) 120.1

C(11)-C(10)-C(9) 120.8(2)

C(11)-C(10)-H(10) 119.6

C(9)-C(10)-H(10) 119.6

O(3)-C(11)-C(10) 123.5(3)

O(3)-C(11)-C(12) 115.6(3)

C(10)-C(11)-C(12) 120.9(2)

C(10)-C(11)-O(3') 131.8(6)

C(12)-C(11)-O(3') 106.1(6)

O(1)-C(12)-C(7) 123.87(19)

O(1)-C(12)-C(11) 118.97(19)

C(7)-C(12)-C(11) 117.1(2)

O(2)-C(13)-C(18) 123.41(18)

O(2)-C(13)-C(14) 119.77(18)

C(18)-C(13)-C(14) 116.82(18)

C(15)-C(14)-O(4) 119.85(19)

C(15)-C(14)-C(13) 121.17(19)

O(4)-C(14)-C(13) 118.88(19)

C(14)-C(15)-C(16) 121.2(2)

C(14)-C(15)-H(15) 119.4

C(16)-C(15)-H(15) 119.4

C(17)-C(16)-C(15) 119.0(2)

C(17)-C(16)-H(16) 120.5

C(15)-C(16)-H(16) 120.5

C(16)-C(17)-C(18) 120.8(2)

C(16)-C(17)-H(17) 119.6

C(18)-C(17)-H(17) 119.6

C(17)-C(18)-C(13) 120.88(18)

C(17)-C(18)-C(19) 118.22(19)

C(13)-C(18)-C(19) 120.65(18)

N(4)-C(19)-C(18) 124.81(19)

N(4)-C(19)-H(19) 117.6

C(18)-C(19)-H(19) 117.6

N(4)-C(20)-C(21) 111.90(17)

N(4)-C(20)-H(20A) 109.2

C(21)-C(20)-H(20A) 109.2

N(4)-C(20)-H(20B) 109.2

C(21)-C(20)-H(20B) 109.2

H(20A)-C(20)-H(20B) 107.9

C(31)-C(21)-C(22) 111.0(2)

C(31)-C(21)-C(32) 108.93(19)

C(22)-C(21)-C(32) 107.6(2)

C(31)-C(21)-C(20) 110.69(18)

C(22)-C(21)-C(20) 112.27(18)

C(32)-C(21)-C(20) 106.05(19)

N(1)-C(22)-C(21) 115.34(18)

N(1)-C(22)-H(22A) 108.4

C(21)-C(22)-H(22A) 108.4

N(1)-C(22)-H(22B) 108.4

C(21)-C(22)-H(22B) 108.4

H(22A)-C(22)-H(22B) 107.5

C(4)-C(23)-H(23A) 109.5

C(4)-C(23)-H(23B) 109.5

H(23A)-C(23)-H(23B) 109.5

C(4)-C(23)-H(23C) 109.5

H(23A)-C(23)-H(23C) 109.5

H(23B)-C(23)-H(23C) 109.5

C(4)-C(24)-H(24A) 109.5

C(4)-C(24)-H(24B) 109.5

H(24A)-C(24)-H(24B) 109.5

C(4)-C(24)-H(24C) 109.5

H(24A)-C(24)-H(24C) 109.5

H(24B)-C(24)-H(24C) 109.5

O(4)-C(28)-C(30) 115(2)

O(4)-C(28)-C(30') 106.6(11)

O(4)-C(28)-C(29') 110.9(6)

C(30)-C(28)-C(29') 110(3)

C(30')-C(28)-C(29') 114.3(19)

O(4)-C(28)-C(29) 97.0(8)

C(30)-C(28)-C(29) 109(4)

C(30')-C(28)-C(29) 110(2)

O(4)-C(28)-H(28) 111.7

C(30)-C(28)-H(28) 111.7

C(30')-C(28)-H(28) 117.3

C(29')-C(28)-H(28) 95.7

C(29)-C(28)-H(28) 111.7

C(28)-C(29)-H(29A) 109.5

C(28)-C(29)-H(29B) 109.5

C(28)-C(29)-H(29C) 109.5

C(28)-C(30)-H(30A) 109.5

C(28)-C(30)-H(30B) 109.5

C(28)-C(30)-H(30C) 109.5

C(28)-C(29')-H(29D) 109.5

C(28)-C(29')-H(29E) 109.5

H(29D)-C(29')-H(29E) 109.5

C(28)-C(29')-H(29F) 109.5

H(29D)-C(29')-H(29F) 109.5

H(29E)-C(29')-H(29F) 109.5

C(28)-C(30')-H(30D) 109.5

C(28)-C(30')-H(30E) 109.5

H(30D)-C(30')-H(30E) 109.5

C(28)-C(30')-H(30F) 109.5

H(30D)-C(30')-H(30F) 109.5

H(30E)-C(30')-H(30F) 109.5

C(21)-C(31)-H(31A) 109.5

C(21)-C(31)-H(31B) 109.5

H(31A)-C(31)-H(31B) 109.5

C(21)-C(31)-H(31C) 109.5

H(31A)-C(31)-H(31C) 109.5

H(31B)-C(31)-H(31C) 109.5

C(21)-C(32)-H(32A) 109.5

C(21)-C(32)-H(32B) 109.5

H(32A)-C(32)-H(32B) 109.5

C(21)-C(32)-H(32C) 109.5

H(32A)-C(32)-H(32C) 109.5

H(32B)-C(32)-H(32C) 109.5

C(11)-O(3)-C(25) 119.6(4)

O(3)-C(25)-C(26) 104.7(4)

O(3)-C(25)-C(27) 110.4(4)

C(26)-C(25)-C(27) 112.4(5)

O(3)-C(25)-H(25) 109.7

C(26)-C(25)-H(25) 109.7

C(27)-C(25)-H(25) 109.7

C(25')-O(3')-C(11) 114.2(11)

O(3')-C(25')-C(26') 108.0(14)

O(3')-C(25')-C(27') 111.3(15)

C(26')-C(25')-C(27') 115.7(16)

O(3')-C(25')-H(25') 107.2

C(26')-C(25')-H(25') 107.2

C(27')-C(25')-H(25') 107.2

C(25')-C(26')-H(26D) 109.5

C(25')-C(26')-H(26E) 109.5

H(26D)-C(26')-H(26E) 109.5

C(25')-C(26')-H(26F) 109.5

H(26D)-C(26')-H(26F) 109.5

H(26E)-C(26')-H(26F) 109.5

C(25')-C(27')-H(27D) 109.5

C(25')-C(27')-H(27E) 109.5

H(27D)-C(27')-H(27E) 109.5

C(25')-C(27')-H(27F) 109.5

H(27D)-C(27')-H(27F) 109.5

H(27E)-C(27')-H(27F) 109.5

_____________________________________________________________

Symmetry transformations used to generate equivalent atoms.
