# Supplementary material for: A Generator-Produced Gallium-68 Radiopharmaceutical for PET Imaging of Myocardial Perfusion
Source: PLoS One. 2014 Oct 29;9(10):e109361. doi: 10.1371/journal.pone.0109361 (PMC4212944; doi:10.1371/journal.pone.0109361)
Supplement: Table S4 — Anisotropic displacement parameters (Å2×103) for [ENBDMP-3-isopropoxy-PI-Ga]+ I− (4). The anisotropic displacement factor exponent takes the form: −2π2[h2 a*2 U11+… +2 h k a* b* U12]. (DOCX) [file pone.0109361.s006.docx]

**Table S4.** Anisotropic displacement parameters (Å^2^ x 10^3^) for [ENBDMP-3-isopropoxy-PI-Ga]^+^ I^-^ **(4)**. The anisotropic displacement factor exponent takes the form: -2π^2^[h^2^ a*^2^ U^11^ + ... + 2 h k a* b* U^12^ ]

______________________________________________________________________

U^11^ U^22^ U^33^ U^23^ U^13^ U^12^

______________________________________________________________________

I(1) 31(1) 59(1) 17(1) -2(1) -1(1) 3(1)

Ga(1) 19(1) 20(1) 13(1) 0(1) -1(1) 4(1)

O(1) 23(1) 33(1) 15(1) -2(1) 1(1) 10(1)

O(2) 18(1) 19(1) 17(1) 2(1) 0(1) 2(1)

O(4) 20(1) 29(1) 30(1) 5(1) 0(1) -7(1)

N(1) 24(1) 32(1) 14(1) 0(1) 0(1) 10(1)

N(2) 30(1) 19(1) 19(1) 4(1) 2(1) 7(1)

N(3) 22(1) 12(1) 17(1) 1(1) -2(1) 1(1)

N(4) 15(1) 26(1) 13(1) -2(1) 0(1) -1(1)

C(1) 37(1) 34(1) 22(1) 8(1) -4(1) 14(1)

C(2) 43(1) 28(1) 20(1) 9(1) 0(1) 8(1)

C(3) 34(1) 16(1) 24(1) 4(1) 2(1) -1(1)

C(4) 32(1) 17(1) 22(1) 1(1) -1(1) -4(1)

C(5) 23(1) 17(1) 18(1) 1(1) -3(1) -4(1)

C(6) 28(1) 12(1) 16(1) 0(1) -4(1) 0(1)

C(7) 28(1) 14(1) 18(1) -1(1) 1(1) 0(1)

C(8) 34(1) 16(1) 20(1) 0(1) 2(1) -2(1)

C(9) 36(1) 21(1) 26(1) 0(1) 9(1) -4(1)

C(10) 27(1) 29(1) 33(1) -6(1) 9(1) -1(1)

C(11) 24(1) 36(1) 26(1) -10(1) 0(1) 5(1)

C(12) 24(1) 21(1) 17(1) -5(1) 2(1) 5(1)

C(13) 18(1) 20(1) 12(1) -1(1) 0(1) 2(1)

C(14) 20(1) 24(1) 18(1) -2(1) 0(1) -3(1)

C(15) 15(1) 34(1) 31(1) 0(1) -2(1) 3(1)

C(16) 25(1) 26(1) 43(1) 6(1) -3(1) 8(1)

C(17) 24(1) 20(1) 33(1) 4(1) 1(1) 0(1)

C(18) 17(1) 20(1) 17(1) -1(1) 0(1) 1(1)

C(19) 20(1) 21(1) 15(1) -1(1) 2(1) -3(1)

C(20) 15(1) 33(1) 20(1) -5(1) 0(1) -1(1)

C(21) 17(1) 44(1) 19(1) -7(1) -4(1) 4(1)

C(22) 21(1) 43(1) 19(1) -2(1) -4(1) 12(1)

C(23) 51(2) 18(1) 25(1) -3(1) 2(1) -1(1)

C(24) 40(1) 28(1) 36(1) 4(1) -6(1) -13(1)

C(28) 26(1) 65(2) 37(1) 26(1) -8(1) -16(1)

C(29) 42(5) 59(2) 55(6) 44(2) 14(4) 4(2)

C(30) 80(4) 70(4) 19(7) 5(6) 11(5) -8(2)

C(29') 42(5) 59(2) 55(6) 44(2) 14(4) 4(2)

C(30') 80(4) 70(4) 19(7) 5(6) 11(5) -8(2)

C(31) 33(1) 44(1) 19(1) -9(1) -2(1) 7(1)

C(32) 21(1) 65(2) 35(1) -10(1) -11(1) 2(1)

O(3) 19(1) 54(3) 21(2) 2(2) 2(1) 7(2)

C(25) 18(1) 78(5) 27(2) -3(2) 4(1) 10(2)

C(26) 31(2) 131(7) 33(2) 7(4) 0(2) 27(4)

C(27) 38(3) 93(5) 48(3) -20(3) 8(2) -21(3)

O(3') 19(1) 54(3) 21(2) 2(2) 2(1) 7(2)

C(25') 18(1) 78(5) 27(2) -3(2) 4(1) 10(2)

C(26') 47(11) 130(17) 22(7) 4(10) -4(6) 53(13)

C(27') 29(7) 101(12) 67(17) -41(12) 16(9) -9(9)

O(1S) 55(1) 35(1) 68(1) -8(1) -1(1) -14(1)

O(2S) 96(11) 59(8) 112(11) 30(8) -13(9) -5(7)

C(1S) 96(11) 59(8) 112(11) 30(8) -13(9) -5(7)

______________________________________________________________________
