# Supplementary material for: A Generator-Produced Gallium-68 Radiopharmaceutical for PET Imaging of Myocardial Perfusion
Source: PLoS One. 2014 Oct 29;9(10):e109361. doi: 10.1371/journal.pone.0109361 (PMC4212944; doi:10.1371/journal.pone.0109361)
Supplement: Table S5 — Hydrogen coordinates (x 104) and isotropic displacement parameters (Å2×103) for [ENBDMP-3-isopropoxy-PI-Ga]+ I− (4). (DOCX) [file pone.0109361.s007.docx]

**Table S5.** Hydrogen coordinates (x 10^4^) and isotropic displacement parameters (Å^2^ x 10^3^) for [ENBDMP-3-isopropoxy-PI-Ga]^+^ I^-^ **(4)**.

______________________________________________________________________

x y z U(eq)

______________________________________________________________________

H(1) 9764(12) 2371(19) 5326(12) 30(7)

H(2) 9966(12) 4190(20) 6480(11) 27(7)

H(1A) 9191 3973 5721 37

H(1B) 9455 3828 5092 37

H(2A) 10423 3874 5452 36

H(2B) 10083 4851 5655 36

H(3A) 10873 4808 6396 30

H(3B) 11086 3721 6235 30

H(5A) 11184 2421 6990 23

H(5B) 11056 2692 7643 23

H(6) 10237 2320 7997 22

H(8) 9418 1807 8543 28

H(9) 8410 1703 8679 33

H(10) 7765 1992 7916 36

H(15) 12372 523 6413 32

H(16) 11910 -955 6670 37

H(17) 10888 -1077 6614 31

H(19) 9958 -442 6282 22

H(20A) 8847 963 6444 27

H(20B) 9063 -59 6174 27

H(22A) 8801 2347 5135 33

H(22B) 8682 2432 5805 33

H(23A) 10656 4322 7898 47

H(23B) 10144 4326 7428 47

H(23C) 10621 5196 7440 47

H(24A) 11855 3806 6948 52

H(24B) 11705 4143 7586 52

H(24C) 11642 4911 7071 52

H(28) 11555 2262 5294 51

H(29A) 11861 3763 5764 78

H(29B) 12141 3690 5139 78

H(29C) 12536 3436 5686 78

H(30A) 12810 1961 5313 85

H(30B) 12389 1861 4768 85

H(30C) 12368 1045 5261 85

H(29D) 11651 3765 5463 78

H(29E) 12099 3515 4956 78

H(29F) 12345 3684 5588 78

H(30D) 12816 1890 5452 85

H(30E) 12487 1797 4851 85

H(30F) 12362 1006 5342 85

H(31A) 9186 771 4726 48

H(31B) 9290 -161 5132 48

H(31C) 9732 759 5159 48

H(32A) 8040 1035 5751 61

H(32B) 8265 -9 5519 61

H(32C) 8166 887 5085 61

H(25) 7289 2658 7286 50

H(26A) 7329 3549 6421 97

H(26B) 6746 2882 6442 97

H(26C) 7302 2537 6070 97

H(27A) 7328 950 6564 90

H(27B) 6814 1202 7009 90

H(27C) 7457 904 7234 90

H(25') 7347 2936 7218 50

H(26D) 7385 4095 6528 100

H(26E) 6806 3443 6423 100

H(26F) 7379 3277 6036 100

H(27D) 7309 1425 6381 99

H(27E) 6780 1697 6805 99

H(27F) 7380 1244 7052 99

H(1S) 5673 932 6399 79

______________________________________________________________________
