# Supplementary material for: A Generator-Produced Gallium-68 Radiopharmaceutical for PET Imaging of Myocardial Perfusion
Source: PLoS One. 2014 Oct 29;9(10):e109361. doi: 10.1371/journal.pone.0109361 (PMC4212944; doi:10.1371/journal.pone.0109361)
Supplement: Table S6 — Torsion angles [°] for [ENBDMP-3-isopropoxy-PI-Ga]+ I− (4). (DOCX) [file pone.0109361.s008.docx]

**Table S6.** Torsion angles [°] for [ENBDMP-3-isopropoxy-PI-Ga]^+^ I^-^ **(4)**.

________________________________________________________________

N(3)-Ga(1)-O(1)-C(12) -35.07(17)

N(4)-Ga(1)-O(1)-C(12) 68.22(17)

N(2)-Ga(1)-O(1)-C(12) -121.94(17)

N(1)-Ga(1)-O(1)-C(12) 154.96(17)

N(3)-Ga(1)-O(2)-C(13) 58.00(15)

N(4)-Ga(1)-O(2)-C(13) -45.27(15)

N(2)-Ga(1)-O(2)-C(13) 144.98(15)

N(1)-Ga(1)-O(2)-C(13) -131.86(15)

O(2)-Ga(1)-N(1)-C(1) -101.65(15)

O(1)-Ga(1)-N(1)-C(1) 80.09(15)

N(3)-Ga(1)-N(1)-C(1) 17.4(5)

N(4)-Ga(1)-N(1)-C(1) 171.73(15)

N(2)-Ga(1)-N(1)-C(1) -14.53(15)

O(2)-Ga(1)-N(1)-C(22) 133.44(15)

O(1)-Ga(1)-N(1)-C(22) -44.82(15)

N(3)-Ga(1)-N(1)-C(22) -107.5(4)

N(4)-Ga(1)-N(1)-C(22) 46.83(15)

N(2)-Ga(1)-N(1)-C(22) -139.44(16)

O(2)-Ga(1)-N(2)-C(3) -45.23(15)

O(1)-Ga(1)-N(2)-C(3) 134.01(15)

N(3)-Ga(1)-N(2)-C(3) 46.58(15)

N(4)-Ga(1)-N(2)-C(3) -106.8(3)

N(1)-Ga(1)-N(2)-C(3) -139.37(15)

O(2)-Ga(1)-N(2)-C(2) 79.29(15)

O(1)-Ga(1)-N(2)-C(2) -101.46(15)

N(3)-Ga(1)-N(2)-C(2) 171.11(15)

N(4)-Ga(1)-N(2)-C(2) 17.8(4)

N(1)-Ga(1)-N(2)-C(2) -14.84(15)

O(2)-Ga(1)-N(3)-C(6) -155.67(16)

O(1)-Ga(1)-N(3)-C(6) 22.45(16)

N(4)-Ga(1)-N(3)-C(6) -68.52(16)

N(2)-Ga(1)-N(3)-C(6) 116.83(16)

N(1)-Ga(1)-N(3)-C(6) 85.1(4)

O(2)-Ga(1)-N(3)-C(5) 35.21(14)

O(1)-Ga(1)-N(3)-C(5) -146.67(14)

N(4)-Ga(1)-N(3)-C(5) 122.36(13)

N(2)-Ga(1)-N(3)-C(5) -52.29(14)

N(1)-Ga(1)-N(3)-C(5) -84.0(4)

O(2)-Ga(1)-N(4)-C(19) 29.29(16)

O(1)-Ga(1)-N(4)-C(19) -149.64(16)

N(3)-Ga(1)-N(4)-C(19) -61.69(17)

N(2)-Ga(1)-N(4)-C(19) 90.9(4)

N(1)-Ga(1)-N(4)-C(19) 123.30(16)

O(2)-Ga(1)-N(4)-C(20) -144.89(14)

O(1)-Ga(1)-N(4)-C(20) 36.18(14)

N(3)-Ga(1)-N(4)-C(20) 124.13(14)

N(2)-Ga(1)-N(4)-C(20) -83.3(4)

N(1)-Ga(1)-N(4)-C(20) -50.88(14)

C(22)-N(1)-C(1)-C(2) 169.43(18)

Ga(1)-N(1)-C(1)-C(2) 41.4(2)

C(3)-N(2)-C(2)-C(1) 169.36(18)

Ga(1)-N(2)-C(2)-C(1) 41.6(2)

N(1)-C(1)-C(2)-N(2) -56.4(2)

C(2)-N(2)-C(3)-C(4) 176.72(18)

Ga(1)-N(2)-C(3)-C(4) -60.6(2)

N(2)-C(3)-C(4)-C(23) -60.3(2)

N(2)-C(3)-C(4)-C(24) -179.77(18)

N(2)-C(3)-C(4)-C(5) 63.6(2)

C(6)-N(3)-C(5)-C(4) -102.5(2)

Ga(1)-N(3)-C(5)-C(4) 67.49(19)

C(3)-C(4)-C(5)-N(3) -64.7(2)

C(23)-C(4)-C(5)-N(3) 60.5(2)

C(24)-C(4)-C(5)-N(3) 179.01(17)

C(5)-N(3)-C(6)-C(7) 165.88(18)

Ga(1)-N(3)-C(6)-C(7) -3.4(3)

N(3)-C(6)-C(7)-C(12) -15.1(3)

N(3)-C(6)-C(7)-C(8) 172.09(19)

C(12)-C(7)-C(8)-C(9) 1.6(3)

C(6)-C(7)-C(8)-C(9) 174.43(19)

C(7)-C(8)-C(9)-C(10) 0.1(3)

C(8)-C(9)-C(10)-C(11) -0.4(3)

C(9)-C(10)-C(11)-O(3) 176.8(4)

C(9)-C(10)-C(11)-C(12) -1.1(4)

C(9)-C(10)-C(11)-O(3') -166.4(10)

Ga(1)-O(1)-C(12)-C(7) 28.9(3)

Ga(1)-O(1)-C(12)-C(11) -153.16(16)

C(8)-C(7)-C(12)-O(1) 175.09(18)

C(6)-C(7)-C(12)-O(1) 2.5(3)

C(8)-C(7)-C(12)-C(11) -2.9(3)

C(6)-C(7)-C(12)-C(11) -175.50(19)

O(3)-C(11)-C(12)-O(1) 6.5(4)

C(10)-C(11)-C(12)-O(1) -175.4(2)

O(3')-C(11)-C(12)-O(1) -6.8(8)

O(3)-C(11)-C(12)-C(7) -175.4(4)

C(10)-C(11)-C(12)-C(7) 2.7(3)

O(3')-C(11)-C(12)-C(7) 171.3(8)

Ga(1)-O(2)-C(13)-C(18) 36.1(2)

Ga(1)-O(2)-C(13)-C(14) -144.44(15)

C(28)-O(4)-C(14)-C(15) 104.3(2)

C(28)-O(4)-C(14)-C(13) -79.3(2)

O(2)-C(13)-C(14)-C(15) 178.87(19)

C(18)-C(13)-C(14)-C(15) -1.6(3)

O(2)-C(13)-C(14)-O(4) 2.5(3)

C(18)-C(13)-C(14)-O(4) -178.02(17)

O(4)-C(14)-C(15)-C(16) 177.9(2)

C(13)-C(14)-C(15)-C(16) 1.5(3)

C(14)-C(15)-C(16)-C(17) 0.4(4)

C(15)-C(16)-C(17)-C(18) -2.1(4)

C(16)-C(17)-C(18)-C(13) 1.9(3)

C(16)-C(17)-C(18)-C(19) 176.2(2)

O(2)-C(13)-C(18)-C(17) 179.44(19)

C(14)-C(13)-C(18)-C(17) -0.1(3)

O(2)-C(13)-C(18)-C(19) 5.3(3)

C(14)-C(13)-C(18)-C(19) -174.25(18)

C(20)-N(4)-C(19)-C(18) 171.39(18)

Ga(1)-N(4)-C(19)-C(18) -2.8(3)

C(17)-C(18)-C(19)-N(4) 163.4(2)

C(13)-C(18)-C(19)-N(4) -22.3(3)

C(19)-N(4)-C(20)-C(21) -108.8(2)

Ga(1)-N(4)-C(20)-C(21) 65.7(2)

N(4)-C(20)-C(21)-C(31) 61.2(2)

N(4)-C(20)-C(21)-C(22) -63.6(2)

N(4)-C(20)-C(21)-C(32) 179.16(19)

C(1)-N(1)-C(22)-C(21) 175.89(19)

Ga(1)-N(1)-C(22)-C(21) -61.2(2)

C(31)-C(21)-C(22)-N(1) -61.2(2)

C(32)-C(21)-C(22)-N(1) 179.69(18)

C(20)-C(21)-C(22)-N(1) 63.4(2)

C(14)-O(4)-C(28)-C(30) -77(4)

C(14)-O(4)-C(28)-C(30') -78(2)

C(14)-O(4)-C(28)-C(29') 156.6(6)

C(14)-O(4)-C(28)-C(29) 167.8(9)

C(10)-C(11)-O(3)-C(25) 1.7(6)

C(12)-C(11)-O(3)-C(25) 179.7(3)

O(3')-C(11)-O(3)-C(25) -125(3)

C(11)-O(3)-C(25)-C(26) 159.6(5)

C(11)-O(3)-C(25)-C(27) -79.2(6)

O(3)-C(11)-O(3')-C(25') 53(2)

C(10)-C(11)-O(3')-C(25') -10.7(19)

C(12)-C(11)-O(3')-C(25') -177.6(13)

C(11)-O(3')-C(25')-C(26') 158.2(16)

C(11)-O(3')-C(25')-C(27') -73.8(19)

________________________________________________________________
