# Supplementary material for: A Generator-Produced Gallium-68 Radiopharmaceutical for PET Imaging of Myocardial Perfusion
Source: PLoS One. 2014 Oct 29;9(10):e109361. doi: 10.1371/journal.pone.0109361 (PMC4212944; doi:10.1371/journal.pone.0109361)
Supplement: Table S7 — Biodistribution Data (%ID/g) for 67Ga-Complex 5a in Sprague Dawley rats (n = 3). (DOCX) [file pone.0109361.s009.docx]

**Table S7.** Biodistribution Data (%ID/g) for ^67^Ga-Complex **5a** in Sprague Dawley rats

(n = 3).

| time(min) P.I. | 5 | | 30 | | 60 | | 120 | |
| --- | --- | --- | --- | --- | --- | --- | --- | --- |
| %ID/g | Mean | SEM | Mean | SEM | Mean | SEM | Mean | SEM |
| blood | 0.18 | 0.02 | 0.04 | 0.00 | 0.03 | 0.00 | 0.01 | 0.00 |
| lung | 0.81 | 0.07 | 0.63 | 0.02 | 0.56 | 0.02 | 0.59 | 0.01 |
| liver | 2.62 | 0.15 | 0.66 | 0.03 | 0.33 | 0.02 | 0.20 | 0.03 |
| kidneys | 8.14 | 0.62 | 5.17 | 0.14 | 4.20 | 0.10 | 3.54 | 0.11 |
| heart | 1.44 | 0.05 | 1.31 | 0.04 | 1.39 | 0.03 | 1.52 | 0.01 |
| brain | 0.02 | 0.00 | 0.02 | 0.00 | 0.02 | 0.00 | 0.02 | 0.00 |
