# Supplementary material for: A Generator-Produced Gallium-68 Radiopharmaceutical for PET Imaging of Myocardial Perfusion
Source: PLoS One. 2014 Oct 29;9(10):e109361. doi: 10.1371/journal.pone.0109361 (PMC4212944; doi:10.1371/journal.pone.0109361)
Supplement: Table S8 — Heart to Tissue Ratio of 67Ga-Complex 5a in Sprague-Dawley rats (n = 3). (DOCX) [file pone.0109361.s010.docx]

**Table S8.** Heart to Tissue Ratio of ^67^Ga-Complex **5a** in Sprague-Dawley rats (n = 3).

| time(min) P.I. | 5 | | 60 | | 120 | |
| --- | --- | --- | --- | --- | --- | --- |
| %ID/g | Mean | SEM | Mean | SEM | Mean | SEM |
| Heart/Blood | 8.39 | 0.90 | 49.68 | 4.28 | 138.83 | 32.60 |
| Heart/Liver | 0.55 | 0.02 | 4.25 | 0.17 | 7.78 | 1.13 |
